# Mitochondrial Genomes of Mammals from the Brazilian Cerrado and Phylogenetic Considerations for the Orders Artiodactyla, Carnivora, and Chiroptera (Chordata: Mammalia)

**DOI:** 10.3390/life14121597

**Published:** 2024-12-03

**Authors:** Luiz Guilherme Pereira Pimentel, Rafael Augusto Silva Soares, Priscila Martins de Assis, Iuri Batista da Silva, Igor Henrique Rodrigues-Oliveira, Renan Rodrigues Rocha, Vinícius Gonçalves de Miranda, Laiena Luz Bassam, Karine Frehner Kavalco, Fabiano Bezerra Menegídio, Caroline Garcia, Rubens Pasa

**Affiliations:** 1 Laboratory of Bioinformatics and Genomics, Federal University of Viçosa, Rio Paranaíba 38810-000, MG, Brazil; luiz.pimentel@ufv.br (L.G.P.P.); kavalco@ufv.br (K.F.K.); 2 Laboratory of Ecological and Evolutionary Genetics, Federal University of Viçosa, Rio Paranaíba 38810-000, MG, Brazil; 3 Institute of Biological Sciences, Federal University of Minas Gerais, Belo Horizonte 31270-901, MG, Brazil; 4 Technological Research Center, University of Mogi das Cruzes, Mogi das Cruzes 08780-911, SP, Brazil; fabianomenegidio@umc.br; 5 Department of Animal Biology, Federal University of Viçosa, Viçosa 36570-900, MG, Brazil; 6 Integrated Biotechnology Center, University of Mogi das Cruzes, Mogi das Cruzes 08780-911, SP, Brazil; 7 Laboratory of Cytogenetics, University of Southeastern of Bahia, Jequié 45205-490, BA, Brazil; caroline.garcia@uesb.edu.br

**Keywords:** mitogenomes, mtDNA, biodiversity, Brazilian savanna, phylogenetics

## Abstract

We assembled and annotated the complete mitochondrial genomes of *Lycalopex vetulus* (hoary fox), *Cerdocyon thous* (bush dog), *Tayassu pecari* (white-lipped peccary), and *Tadarida brasiliensis* (Brazilian free-tailed bat). The mitogenomes exhibited typical vertebrate structures, containing 13 protein-coding genes, 22 tRNA genes, 2 ribosomal RNA genes, and a D-loop region. Phylogenetic reconstruction using the 13 protein-coding genes revealed robust relationships among species within Carnivora, Chiroptera, and Artiodactyla, corroborating previous studies. Secondary structure analysis of tRNAs and ribosomal genes showed slight variations among species of the same order. This research highlights the importance of mitochondrial genomics in understanding the evolutionary relationships and genetic diversity of Cerrado mammals, contributing to conservation efforts for this unique ecosystem.

## 1. Introduction

**Figure 1:**
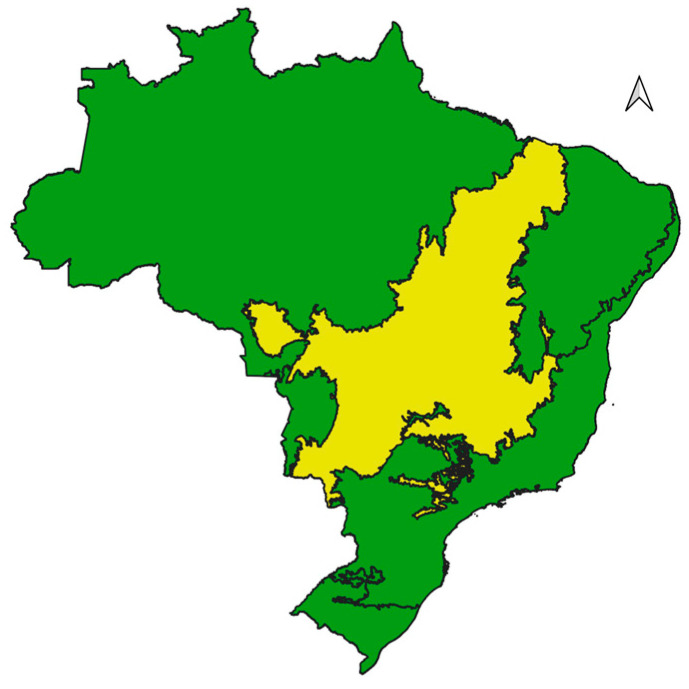
Illustration of a map of Brazil (green) showing the distribution of the Cerrado in yellow. The arrowhead indicates north.

The Brazilian Cerrado is one of the largest tropical savanna ecosystems in the world, covering approximately 25% of the national territory and parts of countries like Bolivia and Paraguay [1,2]. In Brazil, the Cerrado spans about 2 million km^2^, ranking as the second largest among the main biomes (Figure 1), and encompasses the states of Goiás, Tocantins, Mato Grosso, Mato Grosso do Sul, Minas Gerais, Bahia, Maranhão, Piauí, Rondônia, Paraná, São Paulo, and the Federal District, as well as enclaves in Amapá, Roraima, and Amazonas [1,3].

The Cerrado’s biodiversity is greatly favored by its location. It houses the headwaters of major South American river basins, including the Paraná–Paraguay, Araguaia–Tocantins, and São Francisco [4]. Additionally, it encompasses the upper catchments of significant Amazon River tributaries, such as the Xingu and Tapajós. This unique positioning ensures the resources for diverse living organisms inhabiting the region [4].

The Cerrado exhibits distinct phytophysiognomies, including “campo limpo” (open pasture), “campo sujo” (sparsely wooded cerrado), “cerradão” (dense forest), and “cerrado stricto sensu” (typical savanna), and boasts the highest flora richness among savannas, with approximately 10,000 native species already catalogued [2,5]. Considered a biodiversity hotspot similar to the Atlantic Forest, the Cerrado possesses up to 1500 plant species; however, it persists with only 34% of its original vegetation [2,5,6].

One of the greatest threats to the biome is the expansion of the agricultural frontier, resulting in the conversion of natural areas into pastures. Land use change alters soil cover, increasing temperatures and reducing humidity [1,3]. This climate alteration tends to trigger anthropogenic fires that pose a risk to local biodiversity, affecting everything from small invertebrates to large mammals. One of the predominant characteristics of the Cerrado is its natural fires during the rainy season, in which the biome faces no threat [7,8].

Of the 751 mammal species described in Brazil [9], approximately 28% reside in the Cerrado, totaling an average of 211 species [9,10]. Considered “Umbrella Species,” they include the *Panthera onca* (Linnaeus, 1758) and the giant river otter *Pteronura brasiliensis* (Zimmermann, 1780), while *Hydrochoerus hydrochaeris* (Linnaeus, 1766) is classified as a “flagship species” [11,12]. Due to their charisma, these species protect others and can be used as cultural emblems, assisting in conservation campaigns. By conserving these species, others less known to the public also benefit [1].

The identification and classification of species play a fundamental role in biological research. While traditional morphological characteristics have been widely used for species delimitation, molecular markers have gained significant prominence recently due to their enhanced precision, objectivity, and ability to handle taxonomic uncertainties [13,14,15]. The identification and classification of species through molecular data, including the mitochondrial genome, have emerged as valuable resources to assist classical taxonomy in effectively elucidating the vast diversity of species.

Despite the conservation of gene composition among vertebrate mitochondrial genomes, which typically consist of 13 protein-coding genes (PCGs), 22 transfer RNA genes (tRNA), and 2 ribosomal RNA genes (rRNA), variations in gene order, tRNA composition, and the presence of repetitive regions in the D-loop have been observed. Some of these variations have been associated with specific taxonomic groups, making studying mitochondrial genomes an intriguing research area, particularly in conducting studies of phylogenetic relationships, biogeography, evolution, and ecology [16,17,18]. Therefore, conducting studies involving assembling and describing mitochondrial genomes is crucial.

A complete mitochondrial genome can be assembled through conventional Sanger sequencing, where overlapping mitochondrial fragments are sequenced and subsequently aligned to construct the circular genome. However, with next-generation sequencing techniques, vast amounts of data encompassing nuclear genomic and mitochondrial sequences can now be generated. Bioinformatics tools have facilitated the identification of mitochondrial sequences and expedited the assembly process, resembling the assembly of a puzzle. Subsequently, annotation is performed to discern the composition and order of mitochondrial genes within the mitogenome [19].

In this study, we present four new complete mitochondrial genomes of mammals living in the Brazilian Cerrado and conduct a comprehensive survey based on the latest lists of mammalian species in Brazil, their respective status on the IUCN’s (International Union for Conservation of Nature) Red List, and the availability of complete mitochondrial sequences in the GenBank. The ultimate goal is to provide valuable contributions and insights to guide future research and conservation efforts related to Cerrado mammals.

## 2. Materials and Methods

### 2.1. Data Collection

We searched the literature, including Fauna Surveys, Biogeography Studies, Lists of Threatened Species, Fauna Inventories, and the Red Book of Threatened Brazilian Fauna, to obtain a list of mammal species distributed in the Cerrado. After compiling the list, we researched the status of each species on the IUCN’s Red List. Each species’ conservation status is defined by five criteria: historical reduction and decline/population fluctuation, geographic distribution and habitat loss, population distribution, and risk of extinction [20]. 

To compile the list of mammal species, we used as a criterion the history of occurrence and documented records in survey studies, fauna lists, and inventories conducted both in the Brazilian Cerrado and in other biomes of the country, such as the Atlantic Forest, Pampas, Caatinga, Pantanal, and Amazon. Next, we searched GenBank for each species’ complete mitochondrial genomes. For species that did not have described mitochondrial genomes, we searched DNA-seq sequencing libraries of these species in the Sequence Read Archive (SRA) repository in the National Center for Biotechnology Information (NCBI) database.

### 2.2. Sample Information

All sequencing data used in this study were collected from the Sequence Read Archive (SRA) public repository. Below, we describe how the authors performed the sequencing. The libraries of *Lycalopex vetulus* and *Cerdocyon thous* were previously prepared, and genomic DNA was sequenced with paired-end 150 bp reads on Illumina HiSeqX or NovaSeq 6000 instruments [21]. Dr Daniel E. Chavez deposited the raw genomic DNA sequencing libraries in the public database of the National Center for Biotechnology Information (NCBI) under the project code PRJNA822671.

In turn, *Tayassu pecari* libraries were prepared using the TruSeq DNA Sample Preparation Kit and sequenced with paired-end 101 bp reads on Illumina HiSeq 2000 instruments [22]. Dr Jaime Gongora from the University of Sydney deposited the raw libraries from the sequencing in the public database of the National Center for Biotechnology Information (NCBI) under the project code PRJNA384704.

Finally, *Tadarida brasiliensis* libraries were of the PACBio HIFI type, and sequencing was performed with the SEQUEL II 2.0 kit. The Broad Institute deposited the raw sequencing libraries in the public database of the National Center for Biotechnology Information (NCBI) under the project code PRJNA399430 [23].

### 2.3. Assembly and Annotation of Mitogenome Sequences

From the raw data libraries of mammal species distributed in the Cerrado found, only four were able to assemble the complete mitochondrial genome, namely *Tayassu pecari* (SRR5651060), *Cerdocyon thous* (SRR18911047), *Tadarida brasiliensis* (SRR7704833), and *Lycalopex vetulus* (SRR18911045). The public libraries with the raw sequencing data were imported into the Galaxy Europe platform [24]. We used the NOVOPlasty V. 4.3.1 tool [25] with a default configuration to assemble the mitochondrial genomes with *cytochrome oxidase B* (*Cyt B*) or the 12S ribosomal gene as seed. We used the MitoAnnotator tool in the MitoFish V.3.89 database [26] to annotate *Tayassu pecari*, *Cerdocyon thous,* and *Tadarida brasiliensis*. As for *Lycalopex vetulus*, the annotation was conducted using Mitoz 3.6 [27].

We used the RNAfold web server [28] to identify the structures of the 22 tRNAs and ribosomal genes (rRNAs) from the mitochondrial genomes of *L. vetulus*, *C. thous*, *T. pecari*, and *T. brasiliensis*. We conducted the Relative Synonymous Codon Usage (RSCU) analysis using the concatenated sequences of the 13 protein-coding genes of the 4 previously stated species using MEGA 11 [29] and an in-house Python and R script [30]. The assembled and annotated mitochondrial genomes are deposited in GenBank under the following codes (to be added with the manuscript’s acceptance): *L. vetulus*, *C. thous*, *T. pecari*, and *T. brasiliensis*.

### 2.4. Phylogenetic Analysis

The statistical methodology employed to reconstruct the phylogenetic tree of all target groups in the present study was Maximum Likelihood. For this, we used the sequences of the 13 protein-coding genes of all species. Subsequently, the genes were individually aligned using the MAFFT V. 7.526 tool on the Galaxy Europe platform [31]. Then, we concatenated the genes using the Concatenator v0.3.6 software [32]. We recovered the phylogeny of three taxa (Carnivora, Artiodactyla, and Chiroptera) using Maximum Likelihood in the IQ-TREE v2.3.6 web server software [33], with parameters set to 10,000 replicates for Ultrafast Bootstrap, Iterations, and Replicates.

## 3. Results and Discussion

### 3.1. Bibliographic Survey of Species

We started with the papers by Marinho-Filho et al. [9] and the update by Gutierrez and Marinho-Filho [10]. However, some of the species in these papers do not occur in the Brazilian Cerrado, such as *Lasiurus borealis* [34], *Pteronotus parnellii* [35], *Lycalopex vetulus* [36], and *Platyrrhinus helleri* [37], among others. Other species marked as endemic are distributed in different biomes, such as *Thrichomys apereoides* [38,39] and *Carterodon sulcidens* [40].

So, we found 205 mammal species in the Cerrado biome [1,4,5,6,10,20,41,42,43,44,45,46], with 14 of them being endemic (Supplementary Material B, Tables S1 and S2). Of these, only 91 have a complete mitochondrial genome available in GenBank (Supplementary Material B, Table S1). However, with the mitochondrial genomes of the four species used in this study, the total will be 95 species, with 110 species lacking a mitochondrial genome available in GenBank. Almost all catalogued species have a conservation status classification assigned by the IUCN or ICMBio (Chico Mendes Institute for Biodiversity Conservation, Ministry of Environment, Brazil). Taking into account the official red list created by the IUCN, most species are classified as Least Concern (LC), with 144 species in this category (70.24% of the total), followed by 7 Near Threatened (NT) (3.41%), 9 Vulnerable (VU) (4.39%), 7 Endangered (EN) (3.41%), two Critically Endangered (CR) (0.97%), one Extinct (EX) (0.48%), and 13 Data Deficient (DD) (6.34%). Among the endemic, the percentages concerning the total species in each conservation category are as follows: LC (30.4%), NT with 4.3%, VU with 4.3%, EN with 17.4%, DD with 30.4%, EX with 4.3%, CR with 0.0%, and NE with 8.7%.

Considering that the Cerrado is the second largest Brazilian biome, covering 24% of the Brazilian territory and hosting a wide diversity of endemic species, the obtained data are alarming indicators that highlight the biome’s great importance and its conservation. It is crucial to emphasize that species with restricted distributions are susceptible to extinction. Factors such as abrupt and drastic changes in habitats, trophic guilds, and ecosystems directly influence the survival of these species [44,47].

Although the survey focused on species endemic to the Cerrado, we also recorded some species with occurrences in other biomes, such as the Atlantic Forest, the Amazon Rainforest, the Pantanal, and the Caatinga. The species with these records are the following: *Thylamys velutinus* (Wagner, 1842), *Thrichomys apereoides* (Lund, 1839), *Trinomys moojeni* (Pessôa, Oliveira & Reis, 1992), and *Lonchophylla bokermanni* (Sazima, Vizotto & Taddei, 1978).

### 3.2. Assembling, Annotation, and Analysis of Mitochondrial Genomes

**Figure 2:**
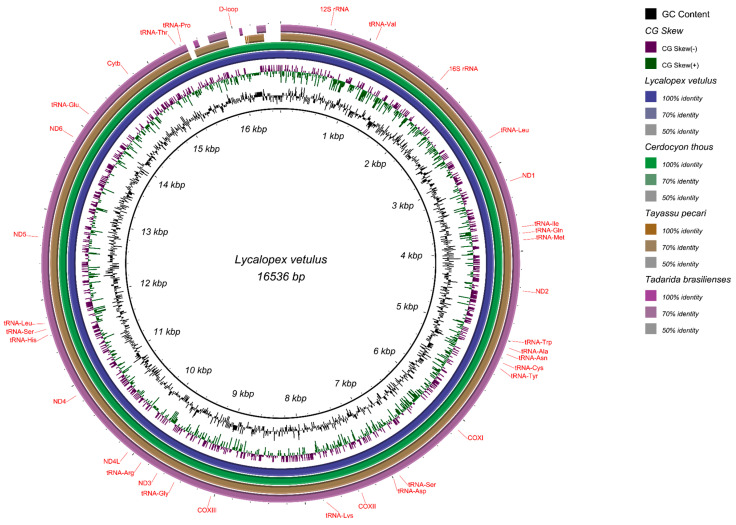
Comparison of the four mitochondrial genomes of cerrado mammals gathered in this study. From the inner to the outer ring, we have the following: GC content in black; GC skew in green and lilac; *L. vetulus* in blue, which was used as the reference mitogenome to generate the BLAST image; *C. thous* in green; *T. pecari* in beige; and *T. brasiliensis* in purple. In the D-loop region, gaps can be observed among the mitogenomes, representing areas with significant differences between the mitochondrial genomes.

About 42.7% of the species occurring in Cerrado have their mitochondrial genomes described. Among the endemics is 21.7% (Supplementary Material B, Table S2). We have found available libraries of four mammal species that lack mitochondrial genomes described and that occur in Cerrado and use them to assemble and annotate the following mitogenomes: *Tayassu pecari* (SRX2888090), *Cerdocyon thous* (SRR18911047), *Tadarida brasiliensis* (SRR7704833), and *Lycalopex vetulus* (SRR18911045). We obtained the mitochondrial genomes of *L. vetulus* with 16,536 bp, *C. thous* with 16,851 bp, *T. pecari* with 16,741 bp, and *T. brasiliensis* with 16,840 bp (Figure 2). 

All species exhibited the expected pattern for vertebrate mitochondrial genomes, including the 13 protein-coding genes (PCGs), 22 transfer RNAs (tRNAs), two ribosomal RNAs (12S and 16S), and the control region (D-loop), The sizes of the mitochondrial genomes were similar and quite close [16]. The GC content also showed slight variation among the species, with values of 37% for *T. brasiliensis*, 38% for *L. vetulus*, 39% for *C. thous*, and 40% for *T. pecari* (Supplementary Materials C and D).

All studied species had 22 tRNAs with a cloverleaf secondary structure, while the *tRNA-SER* exhibited a different structure from the conventional one. Other authors observed the same situation, indicating a pattern in some metazoans’ tRNAs (Supplementary Material A, Figures S1–S4) [48,49].

We can observe that the size variation of the *ribosomal 12S* subunit genes was not significant, with the 12S gene for *L. vetulus* being 956 bp, *C. thous* 955 bp, *T. pecari* 950 bp, and *T. brasiliensis* 965 bp. The *ribosomal 16S* subunit gene also showed slight size variation, with 1578 bp for *L. vetulus*, 1579 bp for *C. thous*, 1569 bp for T. pecari, and 1574 bp for *T. brasiliensis*. Using the RNAfold tool, we obtained the secondary structures of the 12S and 16S genes from the four species. The structures of the 12S and 16S genes for *L. vetulus* and *C. thous* are virtually identical for both species. However, *T. brasiliensis* and *T. pecari* exhibit a secondary structure of the 12S and 16S genes that differs from all other target species in the study. All secondary structures exhibit high complexity, featuring helices, hairpin loops, and loops with many branches, pockets, and protrusions. *T. brasiliensis* and *T. pecari* show a more significant number of branches and pockets in both genes than *L. vetulus* and *C. thous*, indicating that the secondary structure of ribosomal genes will vary according to the group under study.

**Figure 3:**
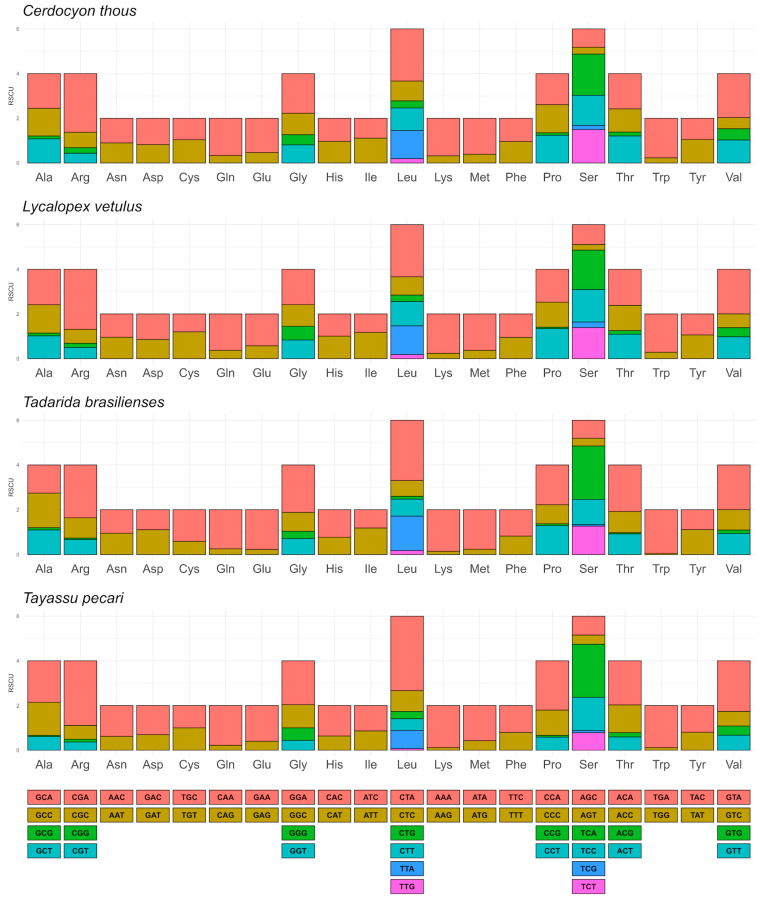
The graph shows the most frequent codons in mitochondrial genes, highlighting the variations in using synonymous codons among the mammalian species analyzed. Codons with RSCU values greater than one are frequently used, indicating a greater preference.

Regarding the RSCU, we observed that the most used codon in the four species was CTA (Leu), with a higher RSCU value in *T. pecari* (3.33), while in *C. thous*, *L. vetulus,* and *T. brasiliensis,* these values were 2.331, 2.33, and 2.69, respectively (Figure 3). The RSCU analysis indicated an overall conserved codon usage, with minor differences between the four species. However, the RSCU was more similar between *C. thous* and *L. vetulus* than the others. This is expected since they are closely related species belonging to Cerdocyonina (Canidae). 

### 3.3. Phylogenetic Analysis

To avoid mistakes due to a large number of valid species but little information about complete mitochondrial genomes to recover a comprehensive phylogeny of mammals, we decided to make the phylogenetic analysis by the order of the species we assembled, using all available information for each one. All species used in the phylogeny reconstruction are described in Supplementary Material B (Tables S3–S5). For *L. vetulus* and *C. thous*, we included 23 mitochondrial genomes from representatives of the order Carnivora (Table S3). For *T. pecari*, we utilized 14 mitogenomes from individuals of the order Artiodactyla (Table S4), while for *T. brasiliensis*, we included 30 mitochondrial genomes of species belonging to the order Chiroptera in the analysis (Table S5).

**Figure 4:**
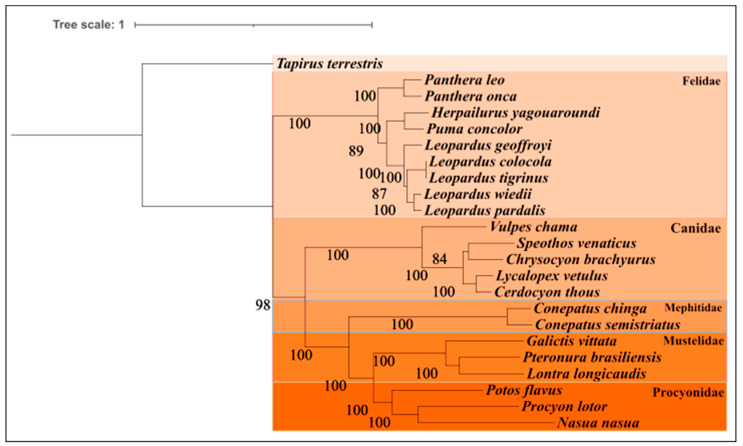
Reconstruction of the phylogenetic tree of the order Carnivora; clades were formed with the families Felidae, Canidae, Mephitidae, Mustalidae, and Procyonidae. *T. terrestris* was used as an outgroup.

The query of the phylogenetic tree of the order Carnivora was partitioned by genes, obtaining a total of 39 partitions, each with a model obtained (Supplementary Material B, Table S6). When analyzing the phylogenetic relationships, we identified five distinct structured clades: Felidae, Canidae, Mephitidae, Mustelidae, and Procyonidae (Figure 4). Remarkably, the species *L. vetulus* and *C. thous* showed a robust relationship, suggesting a sisterhood between these two genera, corroborating previous findings in phylogenetic reconstructions from other studies conducted with both groups [50,51]. Among the other Cerdocyonina canids used in the phylogeny, we recovered a clade composed of *Speothos venaticus* and *Chrysocyon brachyurus* (bootstrap value: 84%), similar to previous studies [50].

However, an additional representative may be necessary to complete the history of this phylogeny. Also noteworthy is the positioning of the species *Vulpes chama* (Vulpini), which emerges as the sister group of Canini in the Canidae family, as pointed out by other phylogenetic studies [51].

**Figure 5:**
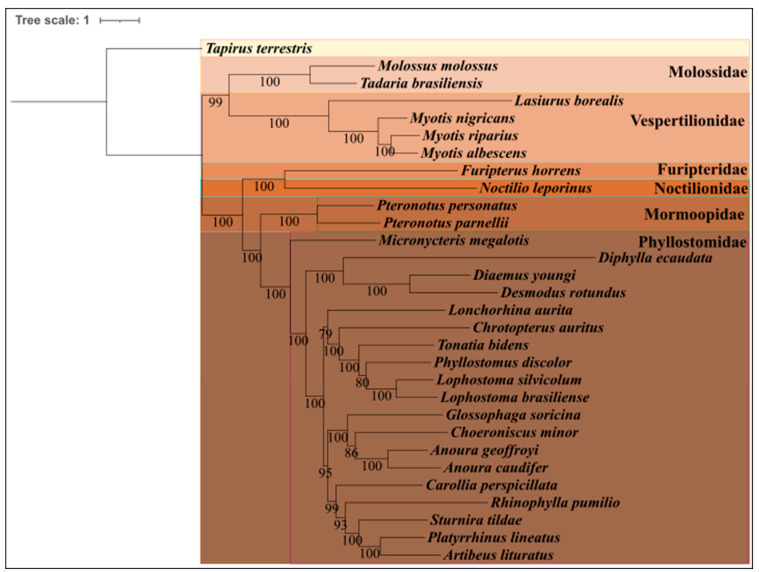
Reconstruction of the phylogenetic tree of the order Chiroptera, clades were formed with the families Molossidae, Vespertilionidae, Furipteridae, Noctilionidae, Mormoopidae, and Phyllostomidae. *Tapirus terrestris* was used as an outgroup.

The reconstruction of the phylogenetic tree of the order Chiroptera was partitioned by genes, obtaining 39 models as the best model for each (Supplementary Material B, Table S7). Upon analyzing the phylogenetic relationships, we identified the presence of six structured clades representing six different families: Molossidae, Vespertilionidae, Furipteridae, Noctilionidae, Mormoopidae, and Phyllostomidae (Figure 5). *T. brasiliensis* and *Molossus molossus* appear as sister species, demonstrating a robust relationship with a reliability of 100%. This grouping among species of the Molossidae family is supported by previous studies, such as that of Gregorin and Cirranello in 2015, highlighting the proximity between these two genera, thus explaining the high reliability observed [52].

Furthermore, upon analyzing the other clades in the phylogenetic tree, we found that almost all groupings exhibit significant reliability, with the lowest reliability recorded at 79% for the alignment of *Lonchorhina aurita* with the other species within the Phyllostomidae family. This lower reliability suggests the possibility of additional representatives that could be included in the Phyllostomidae family clade to improve the accuracy of the phylogeny [53].

In contrast to the present study, the work by Lamb et al. (2011), which also used the mitochondrial gene *Cytochrome Oxidase B* and nuclear genes such as *Histone 3* and *Rag2* in the phylogenetic reconstruction with a methodology similar to the one adopted here, yielded different results. In Lamb et al. phylogeny, *T. brasiliensis* did not form a monophyletic clade with other species of the genus, such as *Tadarida fulminans*. Instead, *T. brasiliensis* appeared as one of the first organisms to diverge within its family, while *T. fulminans* seems to have diverged later in its evolutionary history [54].

**Figure 6:**
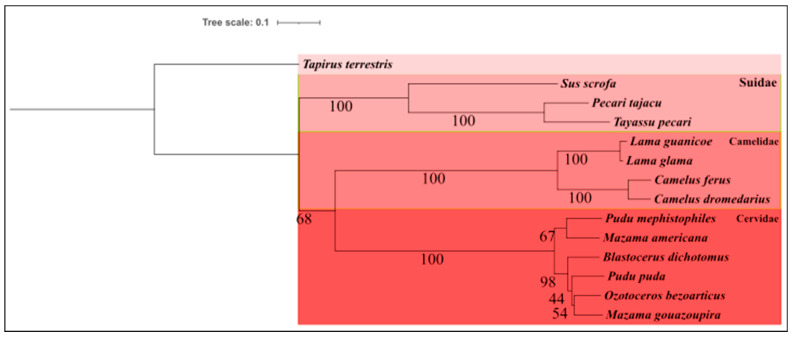
Reconstruction of the phylogenetic tree of the order Artiodactyla; clades were formed with the families Suidae, Camelidae, and Cervidae. *Tapirus terrestris* was used as an outgroup.

The reconstruction of the phylogenetic tree of the order Artiodactyla was partitioned by genes, obtaining 39 models as the best model for each (Supplementary Material B, Table S8). We identified three distinct families through analyses: Suidae, Camelidae, and Cervidae. Notably, the focal species of the group, *T. pecari*, demonstrated a highly reliable grouping with *Pecari tajacu* (Figure 6). This relationship is consistent with previous findings, such as the study by Parisi Dutra et al., 2017, which revealed that *T. pecari* and *P. tajacu* are sister groups, justifying the high reliability observed [55]. Additionally, it is worth noting that the grouping of *Sus scrofa* with *T. pecari* and *P. tajacu* also shows high reliability, suggesting a close relationship among the three species. *S. scrofa* emerges as the sister group of all other species of the Suidae family in Artiodactyla, adding an exciting aspect to understanding phylogenetic relationships within the group [56].

In contrast to the present study, the work by Zurano et al. (2019), which also used mitochondrial genome genes for phylogenetic reconstruction with a methodology similar to the one adopted here, yielded distinct results. In their phylogeny, *T. pecari* and *P. tajacu* are not aligned as closely related sister genera. In Zurano et al.’s study, *T. pecari* formed a clade with *Parachoerus wagneri* (previously *Catagonus wagner*), a species not included in the present study due to its absence in Brazil. Meanwhile, *P. tajacu* is still part of the clade formed by *T. pecari*, but it was the first to diverge within this clade that includes *T. pecari* and *P. wagneri* [56].

## 4. Conclusions

The analysis of the studied species’ mitochondrial genomes revealed notable similarities in size, composition, and nucleotide proportion despite their taxonomic differences. These findings underscore the importance of mitochondrial genomes in understanding the molecular evolution of metazoans, even though the results had shown conservative trends. Further studies exploring additional data sources, such as nuclear analysis, can complement the natural history of the group in this outstanding Brazilian biome, mainly if applied to population studies, aiding in planning conservation strategies.

## Data Availability

The mitochondrial genomes assembled in this study will be available in the Third Party Annotation Section of the DDBJ/ENA/GenBank databases in the future under a TPA accession number. The GenBankvouchers are available in the Supplementary Material D. All supplementary data are available in the article Supplementary Materials A and B.

## Supplementary Materials

The following supporting information can be downloaded at: https://www.mdpi.com/article/10.3390/life14121597/s1.
