# Supplementary material for: Mitochondrial Genomes of Mammals from the Brazilian Cerrado and Phylogenetic Considerations for the Orders Artiodactyla, Carnivora, and Chiroptera (Chordata: Mammalia)
Source: Life (Basel). 2024 Dec 3;14(12):1597. doi: 10.3390/life14121597 (PMC11676698; doi:10.3390/life14121597)
Supplement: Supplementary file 1 [file life-14-01597-s001.zip › Supplementary Material A.pdf]

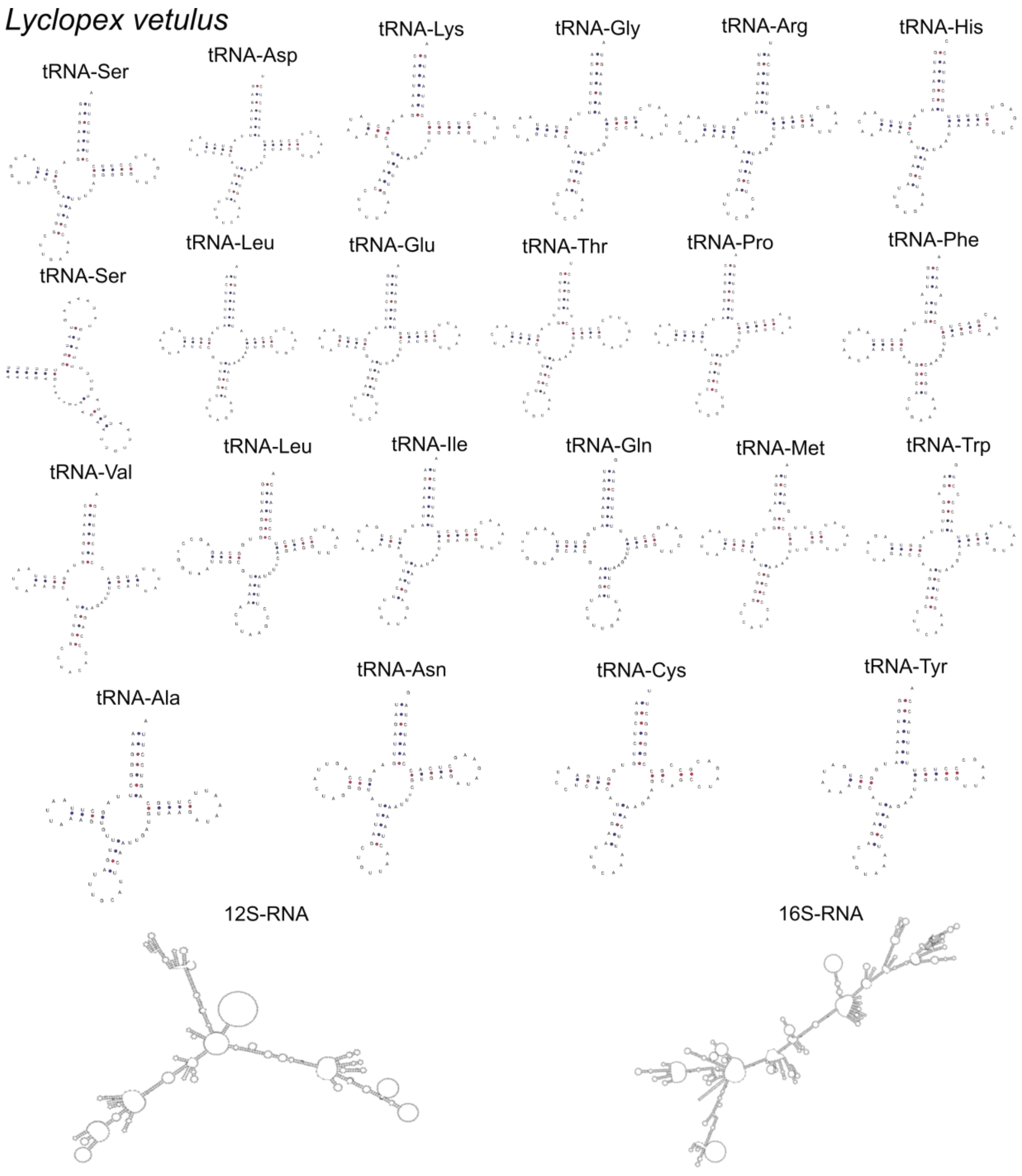

Figure - S1: Secondary structure of tRNAs, 16S rRNA and 12S rRNA of *Lycalopex vetulus*.

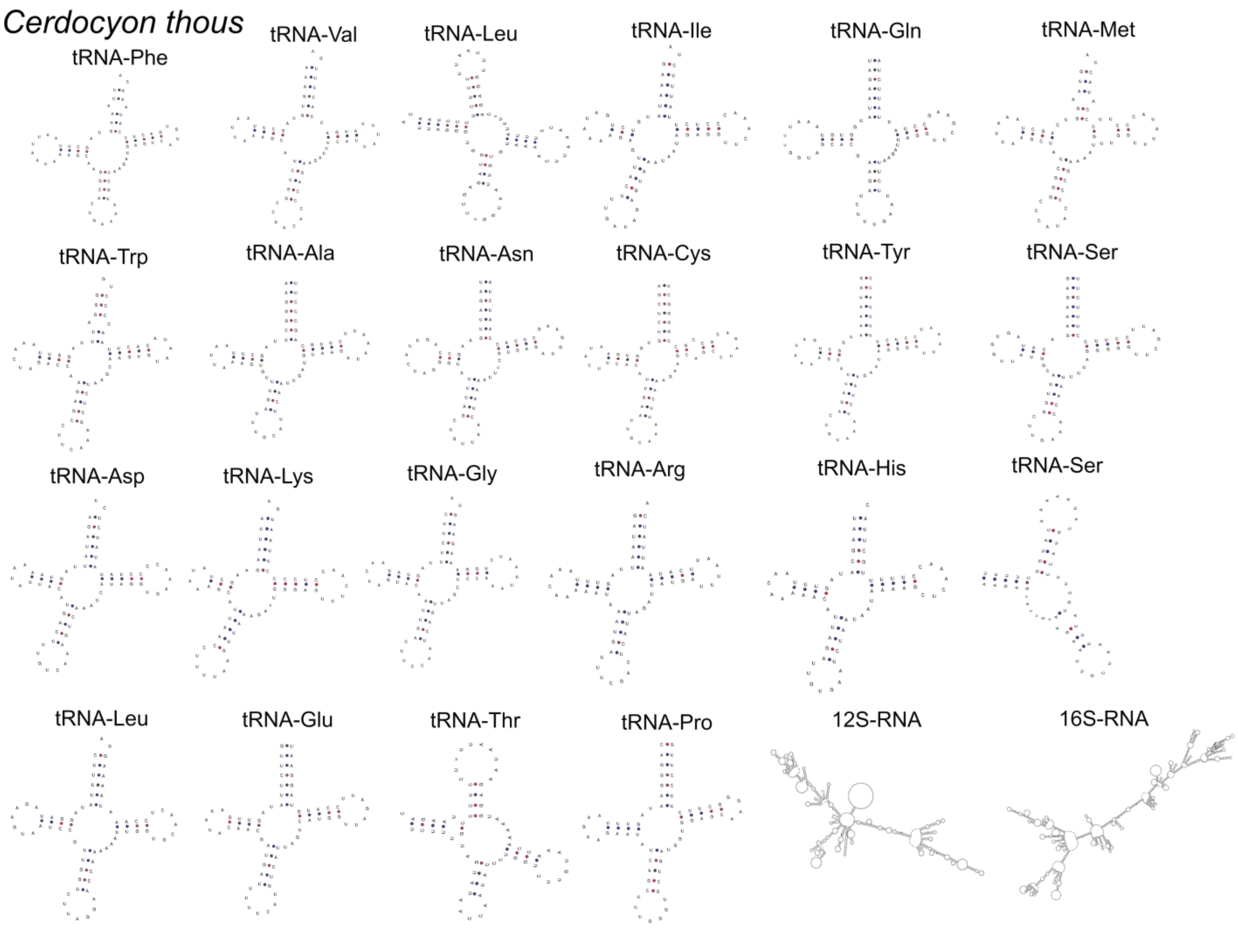

Figure - S2: Secondary structure of tRNAs, 16S rRNA and 12S rRNA of *Cerdocyon thous*.

Tayassu pecari

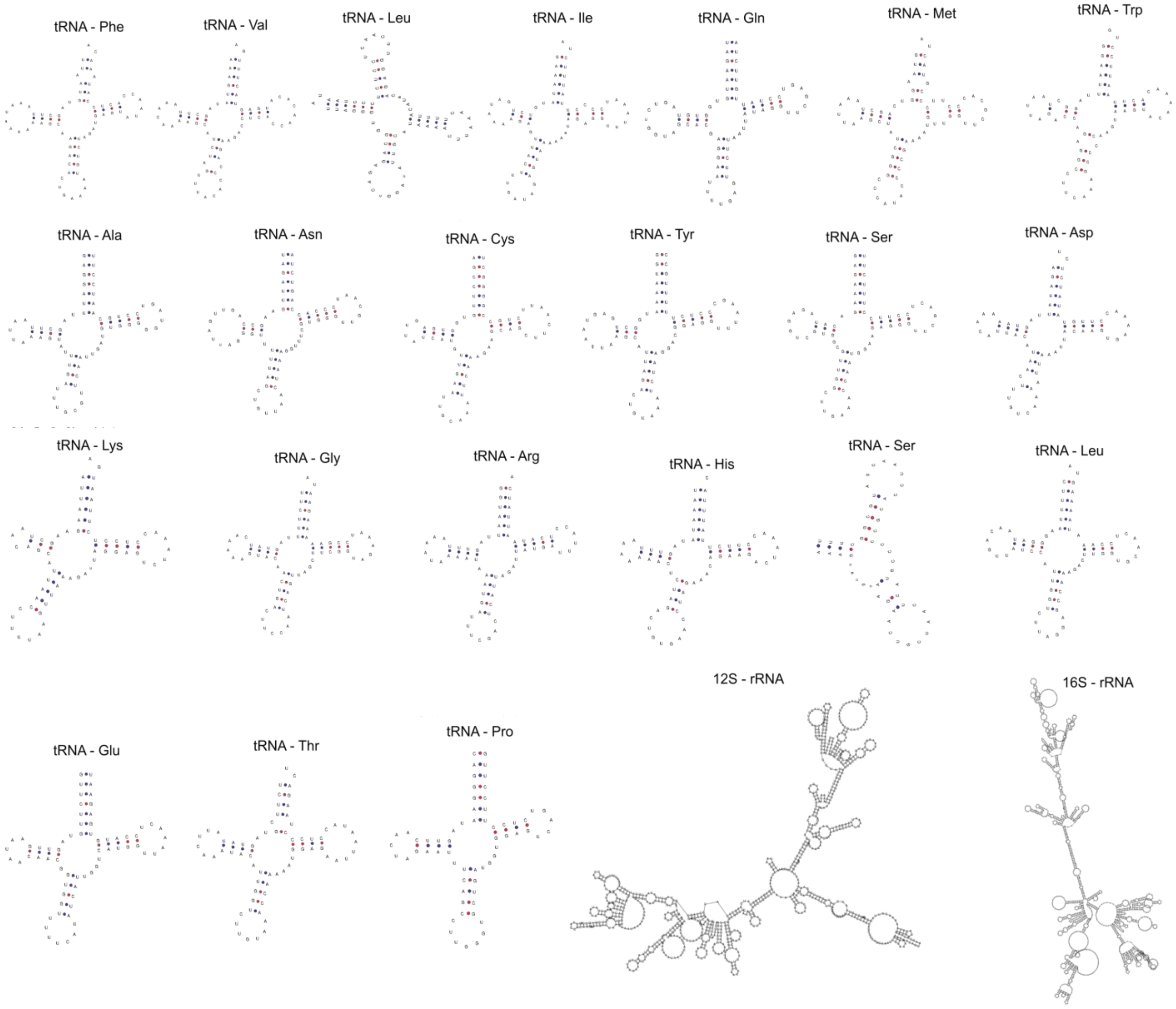

Figure - S3: Secondary structure of tRNAs, 16S rRNA and 12S rRNA of *Tayassu pecari*.

Tadarida brasilienses

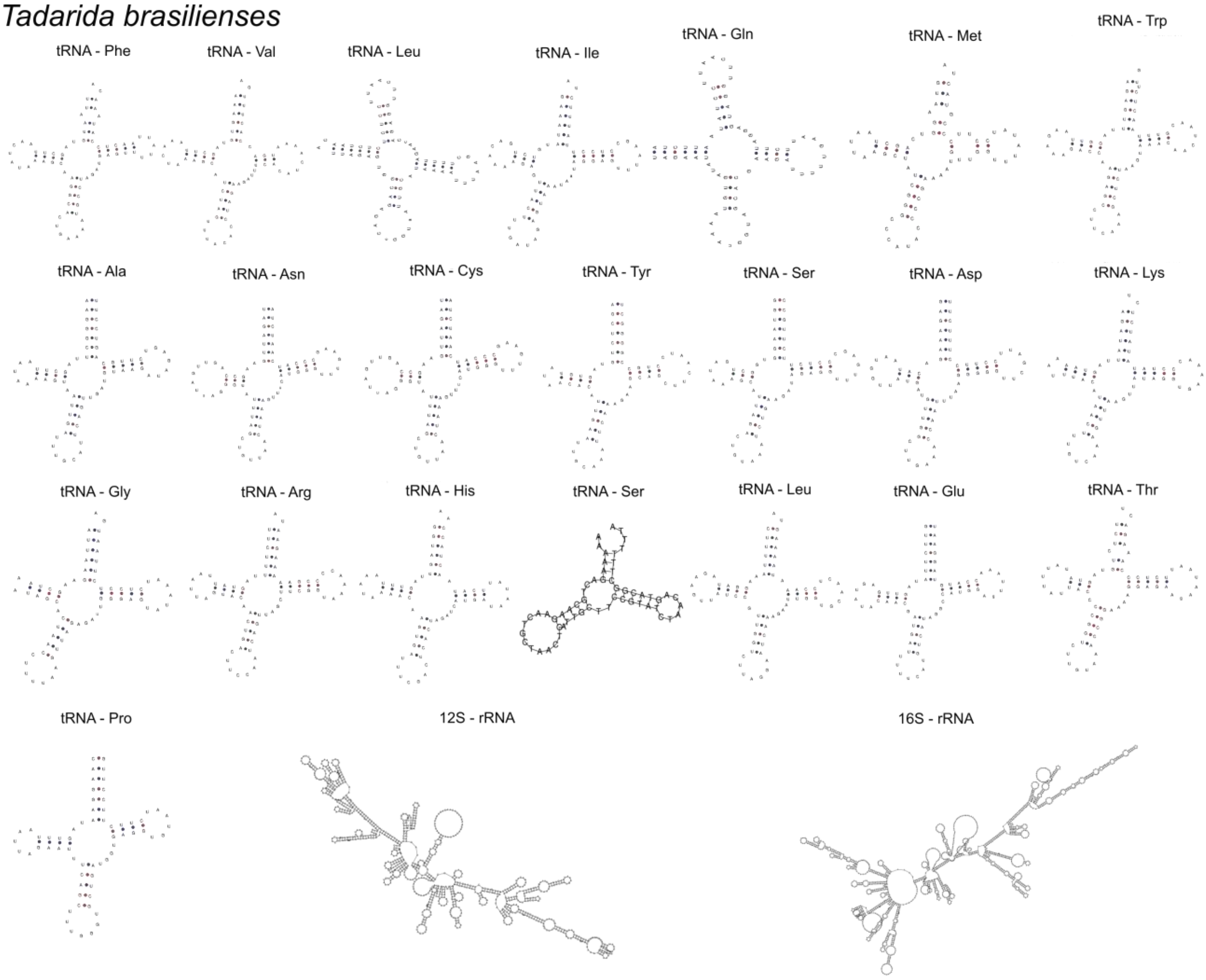

Figure - S4: Secondary structure of tRNAs, 16S rRNA and 12S rRNA of *Tadaria brasilienses*.
