# Supplementary material for: Mitochondrial Genomes of Mammals from the Brazilian Cerrado and Phylogenetic Considerations for the Orders Artiodactyla, Carnivora, and Chiroptera (Chordata: Mammalia)
Source: Life (Basel). 2024 Dec 3;14(12):1597. doi: 10.3390/life14121597 (PMC11676698; doi:10.3390/life14121597)
Supplement: Supplementary file 1 [file life-14-01597-s001.zip › Supplementary Material B.pdf]

Table S1 – List of species occurring in Brazilian Cerrado, its conservation status in the IUCN Red List and the ICMBio Red List, and access to GenBank of mitochondrial genomes when available.

Family	Species	Status and Year of IUCN publication	Status and Year of Brazilian Oficial List	GenBank access
Bradypodidae	*Bradypus variegatus* (Schinz, 1825)	LC (2022)	LC (2018)	NC_028501.1
Callithrichidae	*Callithrix penicillata* (É. Geoffroy Saint-Hilaire, 1812)	LC (2015)	LC (2019)	NC_030788.1
Callithrichidae	*Mico melanurus* (É. Geoffroy in Humboldt, 1812)	LC (2016)	LC (2019)	-
Canidae	*Cerdocyon thous* (Linnaeus, 1766)	LC (2015)	LC (2018)	-
Canidae	*Chrysocyon brachyurus* (Illiger, 1815)	NT (2015)	VU (2023)	NC_024172.1
Canidae	*Lycalopex vetulus* (Lund, 1842)	NT (2019)	VU (2023)	-
Canidae	*Speothos venaticus* (Lund, 1842)	NT (2011)	VU (2023)	NC_053974.1
Caviidae	*Cavia aperea* (Erxleben, 1777)	LC (2016)	LC (2020)	NC_046949.1
Caviidae	*Galea spixii* (Wagler, 1831)	LC (2016)	LC (2021)	-
Caviidae	*Kerodon acrobata* (Moojen, Locks & Langguth, 1997)*	DD (2016)	VU (2023)	-
Cebidae	*Alouatta caraya* (Humboldt, 1812)	NT (2015)	NT (2012)	NC_064185.1
Cebidae	*Aotus infulatus* (Kühl, 1820)	-	LC (2012)	KC592390.1
Cebidae	*Sapajus apella* (Linnaeus, 1758)	LC (2015)	LC (2019)	NC_064167.1
Cebidae	*Sapajus libidinosus* (Spix, 1823)	NT (2015)	NT (2012)	NC_087899.1
Cervidae	*Blastocerus dichotomus* (Illiger, 1815)	VU (2016)	VU (2023)	NC_020682.1
Cervidae	*Mazama americana* (Erxleben, 1777)	DD (2015)	DD (2018)	NC_020719.1
Cervidae	*Subulo gouazoubira* (Smith,1827)	LC (2015)	LC (2018)	NC_020720.1
Cervidae	*Ozotoceros bezoarticus* (Linnaeus, 1758)	NT (2015)	VU (2023)	NC_020766.1
Chlamyphoridae	*Euphractus sexcinctus* (Linnaeus, 1758)	LC (2013)	LC (2018)	NC_028571.1
Chlamyphoridae	*Tolypeutes tricinctus* (Linnaeus, 1758)	VU (2013)	EN (2023)	NC_028576.1
Cricetidae	*Akodon cursor* (Winge, 1887)	LC (2016)	LC (2020)	-
Cricetidae	*Akodon lindberghi* (Hershkovitz, 1990)	DD (2016)	LC (2021)	-
Cricetidae	*Akodon montensis* (Thomas, 1913)	LC (2016)	LC (2020)	NC_025746.1
Cricetidae	*Bibimys labiosus* (Winge, 1887)	LC (2016)	LC (2021)	-
Cricetidae	*Calassomys apicalis* (Pardiñas, Lessa, Teta, Salazar-Bravo & Camara, 2014)*	-	NT (2021)	-
Cricetidae	*Calomys callosus* (Rengger, 1830)	LC (2016)	LC (2020)	-
Cricetidae	*Calomys expulsus* (Lund, 1840)	LC (2016)	LC (2021)	-
Cricetidae	*Calomys laucha* (G. Fischer, 1814)	LC (2016)	LC (2020)	-
Cricetidae	*Calomys tener* (Winge, 1887)	LC (2016)	LC (2020)	-

Cricetidae	*Calomys tocantinsi* (Bonvicino, Lima & Almeida, 2003)	LC (2016)	LC (2020)	-
Cricetidae	*Cerradomys marinhus* (Bonvicino, 2003)	LC (2017)	LC (2020)	-
Cricetidae	*Cerradomys subflavus* (Wagner, 1842)	LC (2016)	LC (2020)	-
Cricetidae	*Oecomys roberti* (Thomas, 1904)	LC (2016)	LC (2021)	NC_065749.1
Cricetidae	*Euryoryzomys lamia* (Thomas, 1901)*	VU (2017)	EN (2023)	-
Cricetidae	*Gyldenstolpia planaltensis* (Avila-Pires, 1972)*	-	EN (2023)	-
Cricetidae	*Holochilus brasiliensis* (Desmarest, 1819)	LC (2016)	LC (2021)	-
Cricetidae	*Holochilus sciureus* (Wagner, 1842)	LC (2016)	LC (2021)	NC_061914.1
Cricetidae	*Juscelinomys candango* (Moojen, 1965)*	EX (2019)	CR (PEX) (2023)	-
Cricetidae	*Kunsia tomentosus* (Lichtenstein, 1830)	CR (2018)	LC (2021)	-
Cricetidae	*Microakodontomys transitorius* (Hershkovitz, 1993)*	EN (2018)	EN (2023)	-
Cricetidae	*Necromys lasiurus* (Lund, 1841)	LC (2016)	LC (2020)	-
Cricetidae	*Nectomys rattus* (Pelzeln, 1883)	LC (2016)	LC (2020)	-
Cricetidae	*Oecomys bicolor* (Tomes, 1860)	LC (2016)	LC (2021)	-
Cricetidae	*Oecomys cleberi* (Locks, 1981)	DD (2019)	LC (2020)	-
Cricetidae	*Oecomys concolor* (Wagner, 1845)	LC (2016)	LC (2021)	-
Cricetidae	*Oligoryzomys moojeni* (Weksler & Bonvicino, 2005)*	DD (2017)	LC (2020)	-
Cricetidae	*Oligoryzomys nigripes* (Olfers, 1818)	LC (2016)	LC (2020)	-
Cricetidae	*Oligoryzomys rupestris* (Weksler & Bonvicino, 2005)*	DD (2017)	EN (2023)	-
Cricetidae	*Oligoryzomys stramineus* (Bonvicino & Weksler, 1998)	LC (2017)	LC (2020)	NC_039723.1
Cricetidae	*Hylaeamys megacephalus* (G. Fischer, 1814)	LC (2016)	LC (2020)	-
Cricetidae	*Oxymycterus delator* (Thomas, 1903)	LC (2016)	LC (2020)	-
Cricetidae	*Pseudoryzomys simplex* (Winge, 1887)	LC (2016)	LC (2020)	-
Cricetidae	*Rhipidomys emiliae* (J. A. Allen, 1916)	LC (2016)	LC (2021)	-
Cricetidae	*Rhipidomys macrurus* (Gervais, 1855)	LC (2016)	LC (2020)	-
Cricetidae	*Sooretamys angouya* (G. Fischer, 1814)	LC (2016)	LC (2020)	-
Cricetidae	*Thalpomys cerradensis* (Hershkovitz, 1990)*	LC (2017)	VU (2023)	-
Cricetidae	*Thalpomys lasiotis* (Thomas, 1916)*	LC (2017)	EN (2023)	-
Cricetidae	*Wiedomys cerradensis* (Gonçalves, Almeida & Bonvicino, 2005)	DD (2017)	LC (2020)	NC_025747.1
Cuniculidae	*Cuniculus paca* (Linnaeus, 1766)	LC (2016)	LC (2021)	NC_079967.1
Cyclopedidae	*Cyclopes didactylus* (Linnaeus, 1758)	LC (2013)	LC (2018)	NC_028564.1
Dasypodidae	*Cabassous tatouay* (Desmarest, 1804)	LC (2013)	LC (2018)	NC_028558.1

Dasypodidae	*Cabassous unicinctus* (Linnaeus, 1758)	LC (2013)	LC (2018)	NC_028559.1
Dasypodidae	*Dasypus novemcinctus* (Linnaeus, 1758)	LC (2013)	LC (2018)	NC_001821.1
Dasypodidae	*Dasypus septemcinctus* (Linnaeus, 1758)	LC (2013)	LC (2018)	NC_028569.1
Dasypodidae	*Priodontes maximus* (Kerr, 1792)	VU (2013)	VU (2023)	NC_028573.1
Dasypodidae	*Tolypeutes matacus* (Desmarest, 1804)	NT (2013)	NT (2018)	NC_028575.1
Dasypodidae	*Tolypeutes tricinctus* (Linnaeus, 1758)	VU (2013)	EN (2023)	NC_028576.1
Dasyproctidae	*Dasyprocta azarae* (Lichtenstein, 1823)	DD (2016)	LC (2021)	-
Didelphidae	*Caluromys lanatus* (Olfers, 1818)	LC (2015)	LC (2019)	-
Didelphidae	*Caluromys philander* (Linnaeus, 1758)	LC (2015)	LC (2019)	-
Didelphidae	*Chironees minimus* (Linnaeus, 1758)		LC (2019)	-
Didelphidae	*Cryptonanus agricolai* (Moojen, 1943)	DD (2016)	LC (2019)	-
Didelphidae	*Didelphis albiventris* (Lund, 1840)	LC (2015)	LC (2019)	-
Didelphidae	*Didelphis aurita* (Wied-Neuwied, 1826)	LC (2015)	LC (2019)	NC_057515.1
Didelphidae	*Didelphis marsupialis* (Linnaeus, 1758)	LC (2016)	LC (2019)	NC_057518.1
Didelphidae	*Gracilinanus agilis (*Burmeister, 1854)	LC (2015)	LC (2019)	NC_054268.1
Didelphidae	*Lutreolina crassicaudata* (Desmarest, 1804)	LC (2016)	LC (2019)	NC_057520.1
Didelphidae	*Marmosa murina* (Linnaeus, 1758)	LC (2015)	LC (2019)	-
Didelphidae	*Marmosops incanus* (Lund, 1840)	LC (2015)	LC (2019)	-
Didelphidae	*Marmosops ocellatus* (Tate, 1931)	LC (2016)	DD (2019)	-
Didelphidae	*Metachirus nudicaudatus* (É. Geoffroy, 1803)	LC (2015)	LC (2019)	NC_006516.1
Didelphidae	*Micoureus constantiae* (Thomas, 1904)	LC (2016)	LC (2019)	-
Didelphidae	*Micoureus demerarae* (Thomas, 1905)	LC (2015)	LC (2019)	-
Didelphidae	*Micoureus paraguayanus* (Tate, 1931)	LC (2015)	LC (2019)	-
Didelphidae	*Monodelphis americana* (Müller, 1776)	LC (2016)	LC (2019)	-
Didelphidae	*Monodelphis domestica* (Wagner, 1842)	LC (2016)	LC (2019)	NC_006299.1
Didelphidae	*Monodelphis kunsi* (Pine, 1975)	LC (2015)	LC (2019)	-
Didelphidae	*Monodelphis rubida*	LC (2016)		-
Didelphidae	*Monodelphis umbristriata* (Müller, 1776)	LC (2015)	LC (2019)	-
Didelphidae	*Monodelphis unistriata* (Wagner, 1842)*	CR (2016)	DD (2019)	-
Didelphidae	*Philander opossum* (Linnaeus, 1758)	LC (2016)	LC (2019)	-
Didelphidae	*Thylamys karimii* (Petter, 1968)	VU (2016)	LC (2019)	-
Didelphidae	*Thylamys macrurus* (Olfers, 1818)	NT (2014)	LC (2019)	-
Didelphidae	*Thylamys velutinus* (Wagner, 1842)	NT (2016)	LC (2019)	-
Echimyidae	*Carterodon sulcidens* (Lund, 1838)*	DD (2016)	DD (2021)	KU892752.1
Echimyidae	*Clyomys bishopi* (Thomas, 1909)	LC (2016)	LC (2020)	-
Echimyidae	*Clyomys laticeps* (Thomas, 1909)	LC (2016)	LC (2020)	KU892753.1
Echimyidae	*Dactylomys dactylinus* (Desmarest, 1817)	LC (2016)	LC (2020)	NC_029876.1

Echimyidae	*Phyllomys brasiliensis* (Lund, 1840)*	EN (2016)	EN (2023)	-
Echimyidae	*Proechimys longicaudatus* (Rengger, 1830)	LC (2016)	LC (2020)	NC_020657.1
Echimyidae	*Proechimys roberti* (Thomas, 1901)	LC (2016)	LC (2020)	NC_039420.1
Echimyidae	*Thrichomys apereoides* (Lund, 1839)	LC (2016)	LC (2020)	KU892773.1
Echimyidae	*Trinomys albispinus* (I. Geoffroy, 1838)	LC (2016)	LC (2021)	KU892761.1
Echimyidae	*Trinomys minor* (Reis & Pessôa, 1995)	-	-	-
Echimyidae	*Trinomys moojeni* (Pessôa, Oliveira & Reis, 1992)	EN (2016)	EN (2023)	KX650080.1
Emballonuridae	*Peropteryx kappleri* (Peters, 1867)	LC (2016)	LC (2018)	-
Emballonuridae	*Peropteryx macrotis* (Wagner, 1843)	LC (2015)	LC (2018)	-
Emballonuridae	*Rhynchonycteris naso* (Wied-Neuwied, 1820)	LC (2016)	LC (2018)	CM073095.1
Emballonuridae	*Saccopteryx bilineata* (Temminck, 1838)	LC (2015)	LC (2018)	CM072282.1
Emballonuridae	*Saccopteryx leptura* (Schreber, 1774)	LC (2015)	LC (2018)	NC_036421.1
Erethizontidae	*Coendou prehensilis* (Linnaeus, 1758)	LC (2016)	NT (2021)	-
Felidae	*Herpailurus yagouaroundi* (É. Geoffroy Saint-Hilaire, 1803)	LC (2014)	VU (2023)	NC_028311.1
Felidae	*Leopardus pardalis* (Linnaeus, 1758)	LC (2014)	LC (2018)	NC_028315.1
Felidae	*Leopardus tigrinus* (Schreber, 1775)	VU (2016)	EN (2023)	NC_028317.1
Felidae	*Leopardus wiedii* (Schinz, 1821)	NT (2014)	VU (2023)	NC_028318.1
Felidae	*Panthera onca* (Linnaeus, 1758)	NT (2016)	VU (2023)	NC_022842.1
Felidae	*Puma concolor* (Linnaeus, 1771)	LC (2014)	NT (2018)	NC_016470.1
Furipteridae	*Furipterus horrens* (Cuvier, 1828)	LC (2016)	VU (2023)	NC_048476.1
Hydrochaeridae	*Hydrochaeris hydrochaeris* (Linnaeus, 1766)	LC (2016)	LC (2020)	BK066995.1
Leporidae	*Sylvilagus brasiliensis* (Linnaeus, 1758)	EN (2018)	DD (2021)	-
Molossidae	*Eptesicus diminutus* (Osgood, 1915)	LC (2016)	LC (2018)	-
Molossidae	*Eptesicus furinalis* (d'Orbigny e Gervais, 1847)	LC (2015)	LC (2018)	-
Molossidae	*Eumops auripendulus* (Shaw, 1800)	LC (2015)	LC (2018)	-
Molossidae	*Eumops bonariensis* (Peters, 1874)	LC (2016)	LC (2018)	-
Molossidae	*Eumops glaucinus* (Wagner, 1843)	LC (2016)	LC (2018)	-
Molossidae	*Eumops hansae* (Sanborn, 1932)	LC (2015)	LC (2018)	-
Molossidae	*Eumops perotis* (Schinz, 1821)	LC (2015)	LC (2018)	-
Molossidae	*Histiotus velatus* (I. Geoffroy, 1824)	DD (2016)	LC (2018)	-
Molossidae	*Lasiurus cinereus* (Palisot de Beauvois, 1796)	LC (2015)	LC (2018)	-
Molossidae	*Lasiurus ega* (Gervais, 1856)	LC (2016)	LC (2018)	-
Molossidae	*Cynomops abrasus* (Temminck, 1826)	DD (2016)	LC (2018)	-

Molossidae	*Neoplatymops mattogrossensis* (Vieira, 1942)	LC (2019)	LC (2018)	-
Molossidae	*Cynomops planirostris* (Peters, 1866)	LC (2015)	LC (2018)	-
Molossidae	*Molossops temminckii* (Burmeister, 1854)	LC (2015)	LC (2018)	-
Molossidae	*Molossus rufus* (É. Geoffroy, 1805)	LC (2015)	LC (2018)	-
Molossidae	*Molossus molossus* (Pallas, 1766)	LC (2015)	LC (2018)	NC_065689.1
Molossidae	*Nyctinomops aurispinosus* (Peale, 1848)	LC (2019)	LC (2018)	-
Molossidae	*Nyctinomops laticaudatus (*É. Geoffroy, 1805)	LC (2015)	LC (2018)	-
Molossidae	*Nyctinomops macrotis* (Gray, 1840)	LC (2015)	LC (2018)	-
Molossidae	*Promops nasutus* (Spix, 1823)	LC (2015)	LC (2018)	-
Molossidae	*Rhogeessa tumida* (Genoways e Baker, 1996)	LC (2016)	LC (2018)	-
Molossidae	*Tadarida brasiliensis* (I. Geoffroy, 1824)	LC (2015)	LC (2018)	CM061282.1
Mormoopidae	*Pteronotus gymnonotus* (Wagner, 1843)	LC (2018)	LC (2018)	-
Mormoopidae	*Pteronotus personatus* (Wagner, 1843)	LC (2016)	LC (2018)	NC_033353.1
Mustelidae	*Eira barbara* (Linnaeus, 1758)	LC (2016)	LC (2018)	-
Mustelidae	*Galictis cuja* (Molina, 1782)	LC (2015)	LC (2018)	-
Mustelidae	*Galictis vittata* (Schreber, 1776)	LC (2015)	LC (2018)	NC_053973.1
Mustelidae	*Lontra longicaudis* (Olfers, 1818)	NT (2020)	LC (2018)	NC_079649.1
Mustelidae	*Pteronura brasiliensis* (Zimmermann, 1780)	EN (2020)	VU (2023)	NC_071787.1
Myrmecophagidae	*Myrmecophaga tridactyla* (Linnaeus, 1758)	VU (2013)	VU (2023)	NC_028572.1
Myrmecophagidae	*Tamandua tetradactyla* (Linnaeus, 1758)	LC (2013)	LC (2018)	NC_004032.1
Natalidae	*Natalus macrourus* (Gervais, 1856)	LC (2008)	VU (2018)	-
Noctilionidae	*Noctilio albiventris* (Desmarest, 1818)	LC (2015)	LC (2018)	-
Noctilionidae	*Noctilio leporinus* (Linnaeus, 1758)	LC (2015)	LC (2018)	NC_037137.1
Phyllostomidae	*Anoura caudifer* (É. Geoffroy, 1818)	LC (2019)	LC (2018)	NC_022420.1
Phyllostomidae	*Anoura geoffroyi* (Gray, 1838)	LC (2016)	LC (2018)	NC_065676.1
Phyllostomidae	*Dermanura cinerea* (Gervais, 1856)	LC (2016)	LC (2018)	-
Phyllostomidae	*Artibeus concolor* (Peters, 1865)	LC(2016)	LC (2018)	-
Phyllostomidae	*Artibeus lituratus* (Olfers, 1818)	LC (2015)	LC (2018)	NC_016871.1
Phyllostomidae	*Artibeus planirostris* (Spix, 1823)	LC (2015)	LC (2018)	-
Phyllostomidae	*Carollia perspicillata* (Linnaeus, 1758))	LC (2015)	LC (2018)	NC_022422.1
Phyllostomidae	*Chiroderma trinitatum* (Goodwin, 1958)	LC (2016)	LC (2018)	-
Phyllostomidae	*Chiroderma villosum* (Peters, 1860)	LC (2015)	LC (2018)	-
Phyllostomidae	*Choeroniscus minor* (Peters, 1868)	LC(2016)	LC (2018)	NC_065683.1
Phyllostomidae	*Chrotopterus auritus* (Peters, 1856)	LC (2015)	LC (2018)	NC_037132.1
Phyllostomidae	*Desmodus rotundus* (É. Geoffroy, 1810)	LC (2015)	LC (2018)	NC_022423.1

Phyllostomidae	*Diaemus youngii* (Jentnik, 1893)	LC (2015)	LC (2018)	NC_037133.1
Phyllostomidae	*Diphylla ecaudata* (Spix, 1823)	LC (2016)	LC (2018)	NC_037138.1
Phyllostomidae	*Glossophaga soricina* (Pallas, 1766)	LC (2015)	LC (2018)	NC_065682.1
Phyllostomidae	*Lonchophylla bokermanni* (Sazima, Vizotto e Taddei, 1978)	EN (2016)	VU (2023)	-
Phyllostomidae	*Lonchophylla dekeyseri* (Taddei, Vizotto e Sazima, 1983)*	EN (2016)	EN (2023)	-
Phyllostomidae	*Lonchorhina aurita* (Tomes, 1863)	LC (2015)	NT (2018)	NC_037135.1
Phyllostomidae	*Macrophyllum macrophyllum* (Schinz, 1821)	LC (2015)	LC (2018)	-
Phyllostomidae	*Glyphonycteris behnii* (Peters, 1865)	DD (2016)	DD (2018)	-
Phyllostomidae	*Micronycteris megalotis* (Gray, 1842)	LC (2015)	LC (2018)	NC_022419.1
Phyllostomidae	*Micronycteris minuta* (Gervais, 1856)	LC (2015)	LC (2018)	-
Phyllostomidae	*Micronycteris sanborni* (Simmons, 1996)	LC (2017)	LC (2018)	-
Phyllostomidae	*Mimon bennettii* (Gray, 1838)	LC (2018)	LC (2018)	-
Phyllostomidae	*Gardnerycteris crenulatum* (É. Geoffroy, 1803)	LC (2018)	LC (2018)	-
Phyllostomidae	*Phylloderma stenops* (Peters, 1865)	LC (2015)	LC (2018)	-
Phyllostomidae	*Phyllostomus discolor* (Wagner, 1843)	LC (2015)	LC (2018)	NC_065690.1
Phyllostomidae	*Phyllostomus elongatus* (É. Geoffroy, 1810)	LC (2015)	LC (2018)	-
Phyllostomidae	*Phyllostomus hastatus* (Pallas, 1767)	LC (2015)	LC (2018)	-
Phyllostomidae	*Platyrrhinus lineatus* (É. Geoffroy, 1810)	LC (2015)	LC (2018)	ON357734.1
Phyllostomidae	*Rhinophylla pumilio* (Peters, 1865)	LC (2015)	LC (2018)	NC_022426.1
Phyllostomidae	*Sturnira lilium* (É. Geoffroy, 1810)	LC (2016)	LC (2018)	-
Phyllostomidae	*Sturnira tildae* (de la Torre, 1959)	LC (2016)	LC (2018)	NC_022427.1
Phyllostomidae	*Tonatia bidens* (Spix, 1823)	DD (2016)	LC (2018)	MZ391834.1
Phyllostomidae	*Lophostoma brasiliense* (Peters, 1866)	LC (2016)	LC (2018)	NC_065678.1
Phyllostomidae	*Lophostoma silvicola* (d'Orbigny, 1836)	LC(2016)	LC (2018)	NC_022424.1
Phyllostomidae	*Trachops cirrhosus* (Spix, 1823)	LC (2015)	LC (2018)	NC_086900.1
Phyllostomidae	*Uroderma bilobatum* (Peters, 1866)	LC (2019)	LC (2018)	-
Phyllostomidae	*Uroderma magnirostrum* (Davis, 1968)	LC (2015)	LC (2018)	-
Phyllostomidae	*Vampyressa pusilla* (Wagner, 1843)	DD (2016)	LC (2018)	-
Procyonidae	*Nasua nasua* (Linnaeus, 1766)	LC (2015)	LC (2018)	NC_020647.1
Procyonidae	*Potos flavus* (Schreber, 1774)	LC (2015)	LC (2018)	NC_053977.1
Procyonidae	*Procyon cancrivorus* (G. Cuvier, 1798)	LC (2015)	LC (2018)	PP999026.1
Tapiridae	*Tapirus terrestris* (Linnaeus, 1758)	VU (2018)	VU (2023)	NC_053962.1
Tayassuidae	*Pecari tajacu* (Linnaeus, 1758)	LC (2011)	LC (2018)	NC_012103.1
Tayassuidae	*Tayassu pecari* (Link, 1795)	VU (2012)	VU (2023)	-
Vespertilionidae	*Eptesicus brasiliensis* (Desmarest, 1819)	LC (2015)	LC (2018)	-

Vespertilionidae	*Myotis albescens* (É. Geoffroy, 1806)	LC (2015)	LC (2018)	NC_036327.1
Vespertilionidae	*Myotis nigricans* (Schinz, 1821)	LC (2019)	LC (2018)	NC_036318.1
Vespertilionidae	*Myotis riparius* (Handley, 1960)	LC (2015)	LC (2018)	NC_036317.1

Table - S2: List of mammal species endemic to the cerrado, their conservation status is presented with data from IUCN and ICMBio, in addition to access to mitogenomes in GenBank.

Family	Species	Status and Year of IUCN publication
Caviidae	*Kerodon acrobata* (Moojen, Locks & Langguth, 1997)	DD (2016)
Cricetidae	*Calassomys apicalis* (Pardiñas, Lessa, Teta, Salazar-Bravo & Camara, 2014)	-
Cricetidae	*Euryoryzomys lamia* (Thomas, 1901)	VU (2017)
Cricetidae	*Gyldenstolpia planaltensis (Ávila-Pires, 1972)*	-
Cricetidae	*Juscelinomys candango (Moojen, 1965)*	EX (2019)
Cricetidae	*Microakodontomys transitorius* (Hershkovitz, 1993)	EN (2018)
Cricetidae	*Oligoryzomys moojeni* (Weksler & Bonvicino, 2005)	DD (2017)
Cricetidae	*Oligoryzomys rupestris* (Weksler & Bonvicino, 2005)	DD (2017)
Cricetidae	*Thalpomys cerradensis* (Hershkovitz, 1990)	LC (2017)
Cricetidae	*Thalpomys lasiotis* (Thomas, 1916)	LC (2017)
Echimyidae	*Carterodon sulcidens (Lund, 1838)*	DD (2016)
Echimyidae	*Phyllomys brasiliensis* (Lund, 1840)	EN (2016)
Didelphidae	*Monodelphis unistriata* (Wagner, 1842)	CR (2016)
Phyllostomidae	*Lonchophylla dekeyseri (Taddei, Vizotto e Sazima, 1983)*	EN (2016)

Table - S3: Species used in the phylogenetic reconstruction of Carnivora, the species that have the marker * after the name, are species from the cerrado.

Carnivora		
**Family**	**Species**	**GenBank access of mitogenome**
Tapiridae	*Tapirus terrestris**	NC_053962
Felidae	*Panthera leo*	KP001495
Felidae	*Panthera onca**	KM236783
Felidae	*Herpailurus yagouaroundi**	KP202279
Felidae	*Puma concolor**	JN999997
Felidae	*Leopardus geoffroyi*	KP202292
Felidae	*Leopardus colocola**	KP202282
Felidae	*Leopardus tigrinus**	FJ550163
Felidae	*Leopardus wiedii*	KP202289
Felidae	*Leopardus pardalis**	MW257208
Canidae	*Vulpes chama*	NC_070063
Canidae	*Speothos venaticus**	MW257226
Canidae	*Chrysocyon brachyurus**	KJ508409
Canidae	*Lycalopex vetulus*	present paper
Canidae	*Cerdocyon thous*	present paper
Mephitidae	*Conepatus chinga*	MH362797
Mephitidae	*Conepatus semistriatus*	MW205849
Mustelidae	*Galictis vittata**	MW257225
Mustelidae	*Pteronura brasiliensis**	OP056176
Mustelidae	*Lontra longicaudis**	OQ658718
Procyonidae	*Potus flavus**	MW257234
Procyonidae	*Procyon lotor*	NC_009126
Procyonidae	*Nasua nasua**	NC_020647

Table - S4: Species used in the phylogenetic reconstruction of Artiodactyla, the species that have the marker * after the name, are species from the cerrado.

**Artiodactyla**		
**Family**	**Species**	**GenBank access of mitogenome**
Camelidae	*Camelus dromedarius*	OM135562
Camelidae	*Camelus ferus*	MH109913
Camelidae	*Lama glama*	AP003426
Camelidae	*Lama guanicoe*	EU681954
Cervidae	*Blastocerus dichotomus**	JN632603
Cervidae	*Mazama americana**	MZ350857
Cervidae	*Mazama gouazoubira**	MZ350862
Cervidae	*Ozotoceros bezoarticus**	MZ350860
Cervidae	*Pudu puda*	NC_020740
Cervidae	*Pudu mephistophiles*	JN632691
Cervidae	*Mazama gouazoubira*	KJ772514
Suidae	*Sus scrofa*	FJ236999
Tayassuidae	*Pecari tajacu**	AP003427
Tayassuidae	*Tayassu pecari**	present paper

Table S5: Species used in the phylogenetic reconstruction of Chiroptera, the species that have the marker * after the name, are species from the cerrado.

Chiroptera		
**Family**	**Species**	**GenBank access of mitogenome**
Tapiridae	*Tapirus terrestris**	NC_053962
Molossidae	*Molossus molossus**	ON653196
Molossidae	*Tadarida brasilienses**	present paper
Molossidae	*Lasiurus borealis*	NC_016873
Molossidae	*Myotis nigricans**	MF143481
Molossidae	*Myotis riparius**	ON357732
Molossidae	*Myotis albescens**	MF143497
Furipteridae	*Furipterus horrens**	MK033190
Noctilionidae	*Noctilio leporinus**	NC_037137
Mormoopidae	*Pteronotus personatus**	KU569221
Mormoopidae	*Pteronotus parnellii**	KF752590
Phyllostomidae	*Micronycteris megalotis**	ON357728
Phyllostomidae	*Diphylla ecaudata**	KU743911
Phyllostomidae	*Diaemus youngi**	KU743906
Phyllostomidae	*Desmodus rotundus**	ON527786
Phyllostomidae	*Lonchorhina aurita**	KU743908
Phyllostomidae	*Chrotopterus auritus**	KU743905
Phyllostomidae	*Tonatia bidens**	MZ391834
Phyllostomidae	*Phyllostomus discolor**	NC_065690
Phyllostomidae	*Lophostoma silvicolum**	HG003311
Phyllostomidae	*Lophostoma brasilienses**	ON310506
Phyllostomidae	*Glossophaga soricina**	ON321893
Phyllostomidae	*Choeroniscus minor**	ON357720
Phyllostomidae	*Anoura geoffroyi**	ON310504
Phyllostomidae	*Anoura caudifer**	NC_022420
Phyllostomidae	*Carollia perspicillata**	HG003309
Phyllostomidae	*Rhinophylla pumilio**	HG003313
Phyllostomidae	*Sturnira tildae**	HG003314
Phyllostomidae	*Platyrrhinus lineatus**	ON357734
Phyllostomidae	*Artibeus lituratus**	JN209840

Table - S6: Substitution models selected by IQ-TREE for partitions in the phylogeny of Carnivora. The table shows the partition ID and the corresponding evolutionary model, with adjustments for variation in substitution rates across sites. Models like TIM3+F+I+G4 and GTR+F+I+G4are the most frequent.

**Carnivora**	
ID	Model
1	ND5_1st: TIM2+F+I+G4
2	ND5_2st: GTR+F+I+G4
3	ND5_3st: TIM3+F+R3
4	Cytb_1st: GTR+F+I+G4
5	Cytb_2st: K3Pu+F+R2
6	Cytb_3st: TPM3+F+R3
7	ATP6_1st: TIM3+F+I+G4
8	ATP6_2st TN+F+R2
9	ATP6_3st: TIM3+F+I+G4
10	ATP8_1st: TN+F+I+G4
11	ATP8_2st: K3Pu+F+I+G4
12	ATP8_3st: TIM3+F+I+G4
13	ND4L_1st: TIM2e+I+G4
14	ND4L_2st: TPM3+F+R2
15	ND4L_3st: K3Pu+F+I+G4
16	ND4_1st: GTR+F+I+G4
17	ND4_2st: TVM+F+I+G4
18	ND4_3st: K3Pu+F+R3
19	ND3_1st: GTR+F+I+G4
20	ND3_2st: TIM3+F+I+G4
21	ND3_3st: K3Pu+F+R2
22	ND2_1st: GTR+F+I+G4
23	ND2_2st:TPM3+F+I+G4
24	ND2_3st: HKY+F+G4
25	ND1_1st: GTR+F+I+G4
26	ND1_2st: TIM+F+I
27	ND1_3st:TPM3+F+R3
28	COX3_1st: TIM3e+G4
29	COX3_2st: TIM2+F+R2
30	COX3_3st: TIM3+F+I+G4
31	COX2_1st: TIM3+F+G4
32	COX2_2st: TPM3+F+R2

33	COX2_3st: TIM3+F+R3
34	COX1_1st: TIM3e+I+G4
35	COX1_2st: TIM2+F+I
36	COX1_3st: TN+F+I+G4
37	ND6_1st: TN+F+G4
38	ND6_2st: TPM3+F+G4
39	ND6_3st: K3Pu+F+I+G4

Table - S7: Substitution models selected by IQ-TREE for partitions in the phylogeny of Chiroptera. The table lists the partition ID and the respective evolutionary model, considering rate variation across sites. Common models like GTR+F+I+G4 and TIM3+F+R3 highlight the range of evolutionary dynamics across different gene regions.

**Chiroptera**	
ID	Model
1	ND5_1st: TIM3+F+R5
2	ND5_2st: GTR+F+I+G4
3	ND5_3st: TVM+F+I+G4
4	Cytb_1st: GTR+F+I+G4
5	Cytb_2st: TVM+F+I+G4
6	Cytb_3st: HKY+F+R5
7	ATP6_1st: TIM3+F+R3
8	ATP6_2st: GTR+F+R3
9	ATP6_3st: TPM3+F+R4
10	ATP8_1st: TIM2+F+I+G4
11	ATP8_2st: HKY+F+R3
12	ATP8_3st: TIM+F+I+G4
13	ND4L_1st: GTR+F+R3
14	ND4L_2st: TIM3+F+I+G4
15	ND4L_3st: TPM3+F+I+G4
16	ND4_1st: GTR+F+R4
17	ND4_2st: TVM+F+I+G4
18	ND4_3st: TIM2+F+R4
19	ND3_1st: SYM+R3
20	ND3_2st: TPM3+F+I+G4
21	ND3_3st: TPM3+F+R4
22	ND2_1st: GTR+F+I+G4
23	ND2_2st: GTR+F+R3
24	ND2_3st: TIM3+F+R3
25	ND1_1st: GTR+F+I+G4
26	ND1_2st: K3Pu+F+R3
27	ND1_3st: HKY+F+R5
28	COX3_1st: SYM+R3
29	COX3_2st: TVM+F+R2
30	COX3_3st: TPM3+F+R4
31	COX2_1st: SYM+I+G4

32	COX2_2st: TIM3+F+I+G4
33	COX2_3st: HKY+F+R4
34	COX1_1st: SYM+I+G4
35	COX1_2st: K3Pu+F+I+G4
36	COX1_3st: TPM2+F+R5
37	ND6_1st: TIM3+F+R3
38	ND6_2st: GTR+F+I+G4
39	ND6_3st: HKY+F+R4

Table - S8: Substitution models selected by IQ-TREE for partitions in the phylogeny of Artiodactyla. The table lists the partition ID and the corresponding evolutionary model, accounting for variations in substitution rates across sites. Models like TIM2+F+I+G4 and TN+F+I+G4 are frequently used, reflecting the diversity of evolutionary patterns in the dataset.

**Artiodactyla**	
ID	Model
1	ND5_1st: TIM2+F+I+G4
2	ND5_2st: TVM+F+I+G4
3	ND5_3st: TN+F+R2
4	Cytb_1st: TIM3e+I
5	Cytb_2st: HKY+F+I
6	Cytb_3st: TN+F+I+G4
7	ATP6_1st: TIM3+F+G4
8	ATP6_2st: TIM3+F+I
9	ATP6_3st: TN+F+I+G4
10	ATP8_1st: TN+F+R3
11	ATP8_2st: TN+F+G4
12	ATP8_3st: TPM3+F+R2
13	ND4L_1st: TIM3e+G4
14	ND4L_2st: HKY+F+I
15	ND4L_3st: TPM3+F+I
16	ND4_1st: GTR+F+G4
17	ND4_2st: TIM2+F+I
18	ND4_3st: TN+F+I+G4
19	ND3_1st: TIM2e+R3
20	ND3_2st: TPM3+F+R2
21	ND3_3st: HKY+F+G4
22	ND2_1st: GTR+F+G4
23	ND2_2st: TPM3+F+I
24	ND2_3st: TIM2+F+R2
25	ND1_1st: TIM2+F+G4
26	ND1_2st: HKY+F+I
27	ND1_3st: TN+F+I+G4
28	COX3_1st: TIM2+F+G4
29	COX3_2st: TIM3+F+I
30	COX3_3st: HKY+F+R3
31	COX2_1st: TIMe+I+G4
32	COX2_2st: TIM3+F+I

33	COX2_3st: TIM+F+I+G4
34	COX1_1st: TIM3e+G4
35	COX1_2st: TPM3+F+I
36	COX1_3st: TN+F+I+G4
37	ND6_1st: GTR+F+R3
38	ND6_2st: HKY+F+G4
39	ND6_3st: HKY+F+R2
