# Supplementary material for: Mitochondrial Genomes of Mammals from the Brazilian Cerrado and Phylogenetic Considerations for the Orders Artiodactyla, Carnivora, and Chiroptera (Chordata: Mammalia)
Source: Life (Basel). 2024 Dec 3;14(12):1597. doi: 10.3390/life14121597 (PMC11676698; doi:10.3390/life14121597)
Supplement: Supplementary file 1 [file life-14-01597-s001.zip › Supplementary Material C.pdf]

Figure - S5: Mitochondrial genome composition of *Cerdocyon thous*. The table presents, in order: gene names, position (bp), start and stop codons, anticodons, and intergenic/overlap regions. In the intergenic/overlap column, positive values indicate distances between genes (intergenic regions), while negative or zero values indicate gene overlaps.

Gene	Posição (pb)	Fita	Códons de Início	Códons de Parada	Anticódon	Região Intergênica/Sobreposta
trnF	1-69	+	-	-	GAA	1
rrnS	70-1024	+	-	-	-	1
trnV	1025-1091	+	-	-	TAC	1
rrnL	1092-2670	+	-	-	-	1
trnL2	2671-2745	+	-	-	TAA	3
nad1	2748-3704	+	ATG	TAA	-	0
trnI	3704-3772	+	-	-	GAT	-2
trnQ	3843-3770	-	-	-	TTG	1
trnM	3845-3914	+	-	-	CAT	1
nad2	3915-4958	+	ATG	TAA	-	-2
trnW	4957-5024	+	-	-	TCA	-19
trnA	5106-5038	-	-	-	TGC	-28
trnN	5179-5108	-	-	-	GTT	-29
trnC	5281-5214	-	-	-	GCA	1
trnY	5348-5282	-	-	-	GTA	1
cox1	5350-6894	+	ATG	TAA	-	-2
trnS2	6960-6892	-	-	-	TGA	7
trnD	6967-7034	+	-	-	GTC	1
cox2	7035-7718	+	ATG	TAA	-	18
trnK	7736-7802	+	-	-	TTT	1
atp8	7804-8007	+	ATG	TAA	-	-42
atp6	7965-8645	+	ATG	TAA	-	0
cox3	8645-9429	+	ATG	TAA	-	0
trnG	9429-9496	+	-	-	TCC	7
nad3	9503-9853	+	ATG	TAA	-	-9
trnR	9844-9912	+	-	-	TCG	1
nad4l	9913-10209	+	ATG	TAA	-	-42
nad4	10251-11631	+	ATG	TAA	-	-50
trnH	11581-11649	+	-	-	CAC	1
trnS1	11650-11709	+	-	-	GCT	1
trnL1	11710-11779	+	-	-	TAG	-9
nad5	11771-13600	+	ATG	TAA	-	-26
nad6	14111-13584	-	ATG	TAA	-	-69
trnE	14180-14112	-	-	-	TTC	5
cob	14185-15324	+	ATG	TAA	-	1
trnT	15325-15394	+	-	-	TGT	-1
trnP	15459-15394	-	-	-	TGG	1
d-loop	15460-16739	+	-	-	-	1

Figure - S6: Composition of the mitochondrial genome of *Lycalopex vetulus*. The table includes, in sequence: gene names, positions (in base pairs), start and stop codons, anticodons, and intergenic/overlap regions. Positive values in the intergenic/overlap column represent distances between genes (intergenic regions), while negative or zero values denote overlapping genes.

Gene	Posição (pb)	Fita	Códons de Início	Códons de Parada	Anticódon	Região Intergênica/Sobreposta
trnF	1-69	+	-	-	GAA	0
rrnS	70-1025	+	-	-	-	0
trnV	1026-1092	+	-	-	TAC	0
rrnL	1093-2670	+	-	-	-	0
trnL2	2671-2745	+	-	-	TAA	2
nad1	2748-3703	+	ATG	TAA	-	0
trnI	3704-3772	+	-	-	GAT	-3
trnQ	3770..3843	-	-	-	TTG	1
trnM	3845..3914	+	-	-	CAT	0
nad2	3915..4956	+	ATG	TAA	-	0
trnW	4957..5024	+	-	-	TCA	0
trnA	5038..5106	-	-	-	TGC	13
trnN	5108..5179	-	-	-	GTT	1
trnC	5214..5280	-	-	-	GCA	34
trnY	5281..5347	-	-	-	GTA	0
cox1	5349..6893	+	ATG	TAA	-	1
trnS2	6891..6959	-	-	-	TGA	-3
trnD	6966..7033	+	-	-	GTC	6
cox2	7034..7717	+	ATG	TAA	-	0
trnK	7735..7801	+	-	-	TTT	17
atp8	7803..8006	+	ATG	TAA	-	1
atp6	7964..8643	+	ATG	TAA	-	-43
cox3	8644..9427	+	ATG	TAA	-	0
trnG	9428..9495	+	-	-	TCC	0
nad3	9496..9842	+	ATG	TAA	-	0
trnR	9843..9911	+	-	-	TCG	0
nad4l	9912..10208	+	ATG	TAA	-	-7
nad4	10202..11579	+	ATG	TAA	-	0
trnH	11580..11648	+	-	-	CAC	0
trnS1	11649..11708	+	-	-	GCT	0
trnL1	11709..11778	+	-	-	TAG	0
nad5	11779..13599	+	ATG	TAA	-	-17
nad6	13583..14110	-	ATG	TAA	-	0
trnE	14111..14179	-	-	-	TTC	4
cob	14184..15323	+	ATG	TAA	-	0
trnT	15324..15392	+	-	-	TGT	-1
trnP	15392..15457	-	-	-	TGG	0
d-loop	15458..16535	+	-	-	-	1

Figure - S7: Mitochondrial genome structure of *Tadarida brasiliensis*. The table lists, in order: gene names, positions (in base pairs), start and stop codons, anticodons, and intergenic/overlap regions. Positive values in the intergenic/overlap column indicate the distance between genes (intergenic regions), while negative or zero values signify gene overlaps.

Gene	Posição (pb)	Fita	Códons de Início	Códons de Parada	Anticódon	Região Intergênica/Sobreposta
trnF	1-69	+	-	-	GAA	1
rrnS	70..1034	+	-	-	-	1
trnV	1035..1103	+	-	-	TAC	1
rrnL	1104..2672	+	-	-	-	1
trnL2	2673..2747	+	-	-	TAA	2
nad1	2750..3705	+	ATG	TAA	-	1
trnI	3706..3774	+	-	-	GAT	-3
trnQ	3772..3844	-	-	-	TTG	-1
trnM	3844..3912	+	-	-	CAT	1
nad2	3913..4954	+	ATG	TAA	-	1
trnW	4955..5022	+	-	-	TCA	4
trnA	5027..5095	-	-	-	TGC	2
trnN	5097..5169	-	-	-	GTT	33
trnC	5202..5267	-	-	-	GCA	1
trnY	5268..5334	-	-	-	GTA	1
cox1	5336..6880	+	ATG	TAA	-	-2
trnS2	6878..6946	-	-	-	TGA	8
trnD	6954..7020	+	-	-	GTC	1
cox2	7021..7704	+	ATG	TAA	-	3
trnK	7708..7774	+	-	-	TTT	1
atp8	7776..7979	+	ATG	TAA	-	-42
atp6	7937..8616	+	ATG	TAA	-	1
cox3	8617..9400	+	ATG	TAA	-	1
trnG	9401..9470	+	-	-	TCC	1
nad3	9471..9818	+	ATG	TAA	-	1
trnR	9819..9886	+	-	-	TCG	1
nad4l	9887..10183	+	ATG	TAA	-	-6
nad4	10177..11554	+	ATG	TAA	-	1
trnH	11555..11622	+	-	-	GTG	1
trnS1	11623..11682	+	-	-	GCT	1
trnL1	11683..11752	+	-	-	TAG	1
nad5	11753..13573	+	ATG	TAA	-	-17
nad6	13557..14084	-	ATG	TAA	-	1
trnE	14085..14154	-	-	-	TTC	4
cob	14159..15298	+	ATG	TAA	-	1
trnT	15299..15368	+	-	-	TGT	1
trnP	15369..15434	-	-	-	TGG	1
d-loop	15435..16839	-	-	-	-	1

Figure - S8: Mitochondrial genome arrangement of *Tayassu pecari*. The table displays, in sequence: gene names, positions (in base pairs), start and stop codons, anticodons, and intergenic/ overlap regions. Positive values in the intergenic/overlap column represent distances between genes (intergenic regions), while negative or zero values indicate overlapping genes.

Gene	Posição (pb)	Fita	Códons de Início	Códons de Parada	Anticódon	Região Intergênica/Sobreposta
trnF	1..69	+	-	-	GAA	1
rrnS	70..1019	+	-	-	-	1
trnV	1020..1088	+	-	-	TAC	1
rrnL	1089..2662	+	-	-	-	1
trnL2	2663..2737	+	-	-	TAA	2
nad1	2740..3694	+	ATG	TAA	-	1
trnI	3695..3763	+	-	-	GAT	-3
trnQ	3761..3833	-	-	-	TTG	1
trnM	3834..3903	+	-	-	CAT	1
nad2	3904..4945	+	ATG	TAA	-	1
trnW	4946..5012	+	-	-	TCA	5
trnA	5018..5085	-	-	-	TGC	1
trnN	5087..5160	-	-	-	GTT	31
trnC	5192..5258	-	-	-	GCA	1
trnY	5259..5329	-	-	-	GTA	1
cox1	5331..6875	+	ATG	TAA	-	-3
trnS2	6879..6947	-	-	-	TGA	6
trnD	6954..7022	+	-	-	GTC	1
cox2	7023..7710	+	ATG	TAA	-	1
trnK	7711..7776	+	-	-	TTT	1
atp8	7778..7981	+	ATG	TAA	-	-43
atp6	7939..8618	+	ATG	TAA	-	1
cox3	8619..9402	+	ATG	TAA	-	1
trnG	9403..9472	+	-	-	TCC	1
nad3	9473..9819	+	ATG	TAA	-	1
trnR	9820..9888	+	-	-	TCG	1
nad4l	9889..10185	+	ATG	TAA	-	-6
nad4	10179..11556	+	ATG	TAA	-	1
trnH	11557..11624	+	-	-	GTG	1
trnS1	11625..11683	+	-	-	GCT	1
trnL1	11684..11753	+	-	-	TAG	1
nad5	11754..13574	+	ATG	TAA	-	-17
nad6	13558..14085	-	ATG	TAA	-	1
trnE	14086..14154	-	-	-	TTC	4
cob	14159..15298	+	ATG	TAA	-	1
trnT	15299..15368	+	-	-	TGT	-1
trnP	15368..15432	-	-	-	TGG	1
d-loop	15433..16740	+	-	-	-	1
