# Supplementary material for: Mitochondrial Genomes of Mammals from the Brazilian Cerrado and Phylogenetic Considerations for the Orders Artiodactyla, Carnivora, and Chiroptera (Chordata: Mammalia)
Source: Life (Basel). 2024 Dec 3;14(12):1597. doi: 10.3390/life14121597 (PMC11676698; doi:10.3390/life14121597)
Supplement: Supplementary file 1 [file life-14-01597-s001.zip › Supplementary Material D.pdf]

Table - S9: the scientific names of the species whose mitochondrial genomes were assembled in the present study, along with the GenBank accession codes of their mitogenomes.

Species	Genbank access
Cerdocyon thous	BK068801
Lycalopex vetulus	BK068802
Tadarida brasiliensis	BK068803
Tayassu pecari	BK068804
